# Evaluation of the Anti-Atopic Dermatitis Effects of α-Boswellic Acid on Tnf-α/Ifn-γ-Stimulated HaCat Cells and DNCB-Induced BALB/c Mice

**DOI:** 10.3390/ijms23179863

**Published:** 2022-08-30

**Authors:** Ya-Chu Tsai, Hsun-Hao Chang, Sheng-Chieh Chou, Thomas W. Chu, Yu-Jou Hsu, Chien-Yu Hsiao, Yuan-Hsin Lo, Nan-Lin Wu, Der-Chen Chang, Chi-Feng Hung

**Affiliations:** 1 Department of Dermatology, Far Eastern Memorial Hospital, New Taipei City 220, Taiwan; 2 School of Medicine, Fu Jen Catholic University, New Taipei City 242, Taiwan; 3 Department of Cardiology, Tainan Municipal Hospital (Managed by Show Chwan Medical Care Corporation), Tainan City 701, Taiwan; 4 Department of Surgery, Taoyuan Armed Force General Hospital, Taoyuan City 325, Taiwan; 5 Department of Surgery, Taoyuan Armed Force General Hospital Hsinchu Branch, Hainchu City 300, Taiwan; 6 Eastern Virginia Medical School, Norfolk, VA 23507, USA; 7 Graduate Institute of Biomedical and Pharmaceutical Science, Fu Jen Catholic University, New Taipei City 242, Taiwan; 8 Research Center for Food and Cosmetic Safety, Research Center for Chinese Herbal Medicine, Chang Gung University of Science and Technology, Taoyuan 333, Taiwan; 9 Department of Dermatology, Fu Jen Catholic University Hospital, Fu Jen Catholic University, New Taipei City 242, Taiwan; 10 Department of Dermatology, MacKay Memorial Hospital, Taipei 104, Taiwan; 11 Department of Mathematics and Statistics, Department of Computer Science, Georgetown University, Washington, DC 20057, USA; 12 School of Pharmacy, Kaohsiung Medical University, Kaohsiung 807, Taiwan

**Keywords:** boswellic acid, atopic dermatitis, TNF-α/IFN-γ, cytokine, dinitrochlorobenzene, nature product

## Abstract

Boswellic acids, triterpenoids derived from the genus *Boswellia* (Burseraceae), are known for their anti-inflammatory and anti-tumor efficacy. Atopic dermatitis is a chronic, non-infectious inflammatory skin disease. However, the effects of α-boswellic acid on atopic dermatitis have not been studied. Therefore, in this study we examined the expression level of pro-inflammatory cytokines, histopathological analysis, and physiological data from BALB/c mice with atopic-like dermatitis induced by 2,4-dinitrochlorobenzene and TNF-α/IFN-γ-stimulated HaCaT cells to better understand the agent’s anti-atopic dermatitis efficacy. First, we found that α-boswellic reduced the epidermal thickening, mast cell numbers, and dermal infiltration of 2,4-dinitrochlorobenzene-induced atopic-like dermatitis in BALB/c mice. Furthermore, we also found that α-boswellic acid can restore transepidermal water loss and skin reddening in mice. In human keratinocytes inflamed by TNF-α/IFN-γ, α-boswellic acid inhibited MAP kinase activation and showed a reduction in NF-κB nuclear translocation. Finally, α-boswellic acid can reduce the expression level of cytokines (IL-1β, IL-6, and IL-8) following the stimulation of TNF-α/IFN-γ in HaCaT cells. Taken together, our study suggests that α-boswellic acids are a potential component for the development of anti-atopic dermatitis drugs.

## 1. Introduction

Frankincense was valued in ancient times as an object used in medicine and worship, especially in Roman Catholic and Orthodox churches, and remains an important balsamic resin [1]. Traditionally, the oleoresin of some *Boswellia* species (such as *Boswellia serrata* and *Boswellia carterii*) has been used in many countries to treat rheumatism and other inflammatory diseases, including Crohn’s disease and ulcerative colitis [2,3]. In addition, extracts and essential oils of frankincense have been used as antiseptics in mouthwashes and in the treatment of cough and asthma [2]. Numerous studies have reported anticancer, anti-inflammatory, immunomodulatory, antibacterial, antiviral, and even antidiabetic activities of several *Boswellia* plants [2,4,5,6,7,8,9,10,11]. India is the most viable source of frankincense. Boswellic acid is one such phytochemical, obtained from the gum resin of *Boswellia* species, which may help treat various chronic diseases. The pharmacological activity of the oleogum resin is mainly due to the pentacyclic boswellic acids, but other compounds also contribute to its bioactivity[1]. Boswellic acid consists of a series of pentacyclic triterpenoid molecules, produced by trees of the genus *Boswellia*, and has been found to be effective against many diseases. The gum resins of *B. serrata* and *B. carterii* contain up to 12 different types of boswellic acid, of which α-boswellic acid is one of the six major acids. It has now been established that boswellic acid is a multi-targeted drug [12]. It regulates a variety of molecular targets, including enzymes, growth factors, kinases, transcription factors, receptors, and other substances involved in cell survival and proliferation [13,14].

The gum resin of *B. serrata* has also been found to be effective in treating various skin problems. Acetyl-11-keto-beta-boswellic acid inhibits the secretion of cytokines by dendritic cells via the TLR7/8 pathway in an imiquimod-induced psoriasis mouse model and in vitro [15]. Boswellic acid acetate induces differentiation and apoptosis in highly metastatic melanoma [16,17].

Atopic dermatitis (AD) is a chronic, non-infectious inflammatory skin disease. One characteristic of AD is persistent itching of the skin. The chronicity of the disease, the course of recurrence, and the financial burden greatly reduce the patients’ and their families’ quality of life. Steroids, antihistamines, and immunosuppressants are currently used to treat AD [18]. However, these drugs have a variety of adverse effects when used long-term or in high doses [19]. While many effective biological agents have been used in atopic dermatitis recently, the associated high costs render their clinical use very limited [19,20]. Therefore, a great unmet medical need is to develop novel, effective, and cost-efficient therapeutics for AD.

Based on past studies on the pharmacological effects of boswellic acids, their anti-inflammatory effects are known. Therefore, we speculate that they must have a considerable degree of potential in managing atopic dermatitis. Since there are no reports in previous studies on the effect of α-boswellic acids in atopic dermatitis, the research in this paper primarily explores whether α-boswellic acid has an effect on atopic dermatitis and studies its mechanism of action.

## 2. Results

### 2.1. Effects of α-Boswellic Acid on 2,4-Dinitrochlorobenzene-Induced Atopic-like Dermatitis in BALB/c Mice

**Figure 1:**
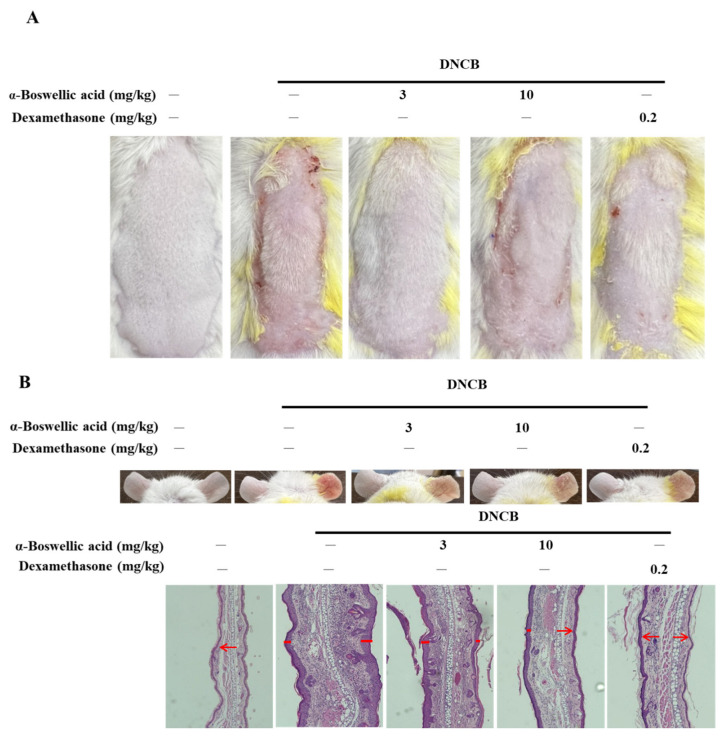

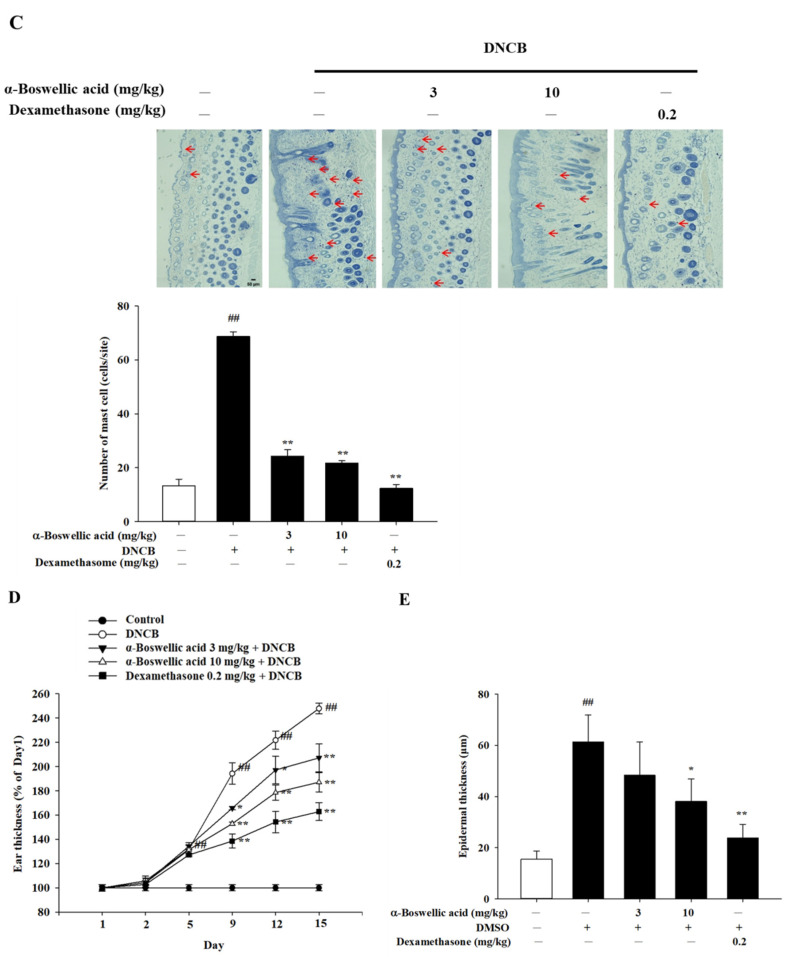
Effects of α-boswellic acid on the histological characteristics of mice with DNCB-induced AD. (**A**) Effect of α-boswellic acid on DNCB-induced atopic dermatitis in dorsal skin. (**B**) Effect of α-boswellic acid on DNCB-induced atopic dermatitis in ears. H&E staining of ear tissue sections. Red arrows point to the epidermis. (**C**) Toluidine blue staining of the dorsal skin; scale bar: 50 μm (upper panel) and statistical analysis of the mast cells (lower panel). The red arrow points to the mast cells. (**D**) Statistical analysis of the ear thickness. (**E**) Histogram of the epidermal thickness. Values represent the mean ± SEM from three independent experiments. * *p* < 0.05 and ** *p* < 0.01 vs. the DNCB-only-treated group; ## *p* < 0.01 vs. the untreated group.

To investigate the effect of α-boswellic acid on AD-like symptoms in BALB/c mice, we sensitized and challenged the skin with DNCB (detailed in the Methods and Materials section). α-Boswellic acid (3 or 10 mg/kg) or dexamethasone (0.2 mg/kg) was administered by intraperitoneal injection 30 min before 2,4-dinitrochlorobenzene (DNCB) challenge on Day 5 to assess its therapeutic potential for AD-like symptoms. On Day 15, the dorsal skin and ears of DNCB-treated BALB/c mice developed severe edema, erythema, erosion, dryness, and skin cracking. The treatment with α-boswellic acid or dexamethasone reduced the severity of the AD-like symptoms (Figure 1A). Furthermore, the epidermis of the DNCB-treated group was significantly thickened. DNCB treatment increased the ear thickness in BALB/c mice (Figure 1B). The ears of DNCB-treated mice were swollen and exhibited epidermal hypertrophy (Figure 1B). We also found that DNCB-induced increased mast cell infiltration in toluidine blue staining compared to the controls (Figure 1C). In contrast, ears of mice treated with α-boswellic acid (5 mg/kg and 10 mg/kg) or dexamethasone (0.2 mg/kg) exhibited less severe epidermal hypertrophy. Treatment with α-boswellic acid or dexamethasone significantly attenuated the increase in ear thickness by DNCB (Figure 1D,E). The efficacy of α-boswellic acid was similar to that of dexamethasone, a well-known steroidal anti-inflammatory agent.

### 2.2. Effects of α-Boswellic Acid on TEWL, Hydration, and Erythema on 2,4-Dinitrochlorobenzene Induced Atopic-like Dermatitis in BALB/c Mice

**Figure 2:**
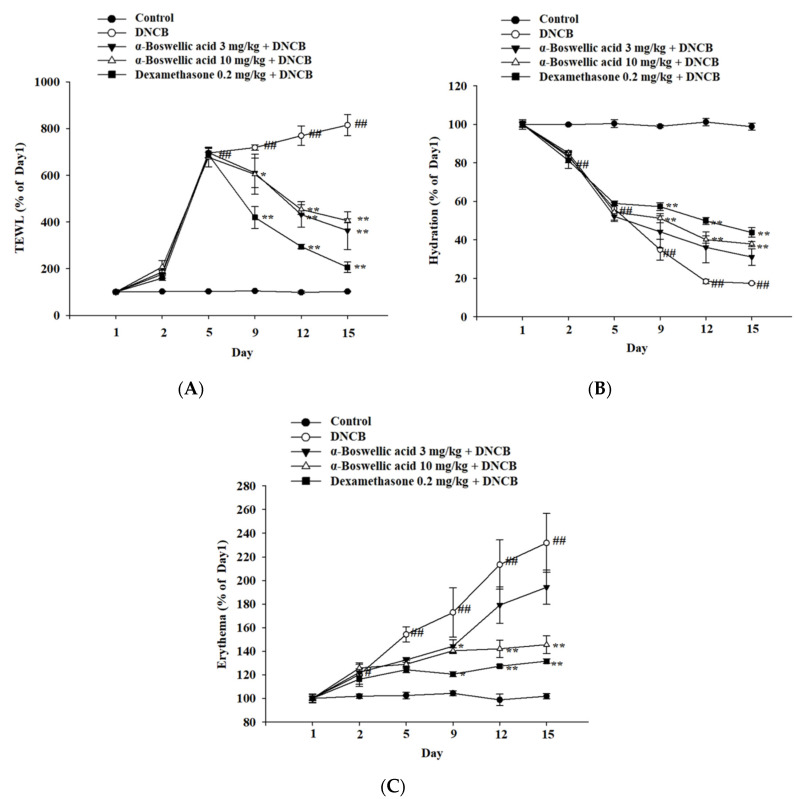
Effects of α-boswellic acid on TEWL, the stratum corneum hydration, and erythema of atopic dermatitis in DNCB-induced mice. Mice were treated with intraperitoneal injection with boswellic acid. (**A**) TEWL; (**B**) stratum corneum hydration; and (**C**) erythema. Values represent the mean ± SEM from three independent experiments. * *p* < 0.05 and ** *p* < 0.01 vs. the DNCB-only-treated group; # *p* < 0.05 and ## *p* < 0.01 vs. the untreated group. AD, atopic dermatitis; DNCB, 2,4-dinitrochlorobenzene; TEWL, transepidermal water loss.

To determine the effect of α-boswellic acid on DNCB-induced skin TEWL in the epidermal layer of mice, TEWL was assessed five times between Days 1 and 15. On the last day of the experiment, increased TEWL was observed in DNCB-induced mice compared to the normal controls. In contrast, treatment with dexamethasone (0.2 mg/kg) and α-boswellic acid (3 mg/kg or 10 mg/kg) significantly decreased cutaneous TEWL in DNCB-treated mice (Figure 2A). To determine the effect of α-boswellic acid on DNCB-induced skin hydration in the epidermal layer of mice, skin hydration was assessed between Day 1 and Day 15. On the last day of the experiment, a significant reduction in skin hydration was observed in DNCB-induced mouse skin compared to the normal controls. In contrast, significant improvements in skin hydration were recorded in DNCB-treated mice treated with dexamethasone (0.2 mg/kg) and α-boswellic acid (3 mg/kg or 10 mg/kg) (Figure 2B). To determine the effect of α-boswellic acid on DNCB-induced erythema in the epidermal layer of mice, erythema was measured between Days 1 and 15. On Days 2, 5, 9, 12, and 15 of the experiment, significantly increased levels of erythema were observed in DNCB-induced mice compared with the normal controls. On Day 5, mice receiving the dexamethasone (0.2 mg/kg) and α-boswellic acid (3 and 10 mg/kg) treatment had significantly decreased erythema (Figure 2C).

### 2.3. Effects of α-Boswellic Acid on TNF-α/IFN-γ-Induced Proinflammatory Cytokines, Chemokines, and Barrier Protein mRNA Expressions in HaCaT Cells

**Figure 3:**
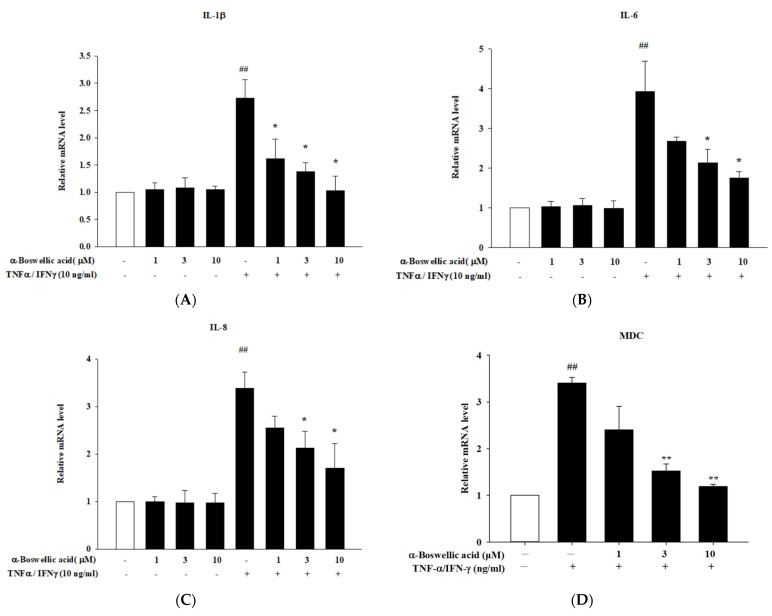

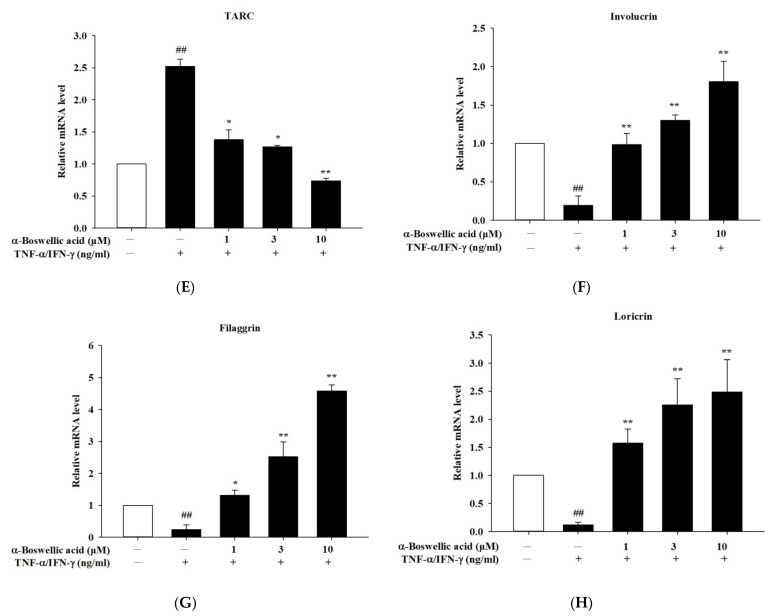
(**A**–**E**) Effects of α-boswellic acid on the TNF-α/IFN-γ-induced proinflammatory cytokines and chemokine mRNA expressions in HaCaT cells. (**F**–**H**) Effects of α-boswellic acid on involucrin, filaggrin, and loricrin mRNA expressions in TNF-α/IFN-γ-induced HaCaT cells. HaCaT cells were pretreated with different concentrations of α-boswellic acid—1, 3, and 10 μM—for 20 min, and then the cells were treated with TNF-α/IFN-γ for 6 h. Total RNA was isolated and the mRNA expression level of IL-1β, IL-6, IL-8, MDC, TARC, involucrin, filaggrin, and loricrin were determined using qPCR. Values represent the mean ± SEM from three or four independent experiments. ## *p* < 0.01 compared with the no-treatment condition; * *p* < 0.05 and ** *p* < 0.01 compared with the only TNF-α/IFN-γ treatment condition.

To study the cytotoxicity of *α-*boswellic on human keratinocyte (HaCaT) cells, the MTT assay was used. *α-*Boswellic did not affect cell viability up to 50 μM for 1 h (data not shown). TNF-α/IFN-γ was used to induce inflammation in HaCaT cells, which is representative of inflammatory skin disease in an in vitro model. We performed real-time PCR to confirm the inhibitory effect of *α-*boswellic on the TNF-α/IFN-γ-induced cytokines and chemokine expression levels in HaCaT cells. We found that the expression levels of IL-1β, IL-6, IL-8, macrophage-derived chemokine (MDC) (CCL22), and thymus and activation-regulated chemokine (TARC) (CCL17) were significantly upregulated by TNF-α/IFN-γ treatment of HaCaT cells. In particular, *α-*boswellic acid treatment significantly decreased IL-1β, IL-6, IL-8, MDC, and TARC mRNA expression (Figure 3A–E). We also showed that the expression of involucrin, filaggrin, and loricrin was decreased by treatment of TNF-α/IFN-γ. As shown in Figure 3F–H, the mRNA expression of involucrin, filaggrin, and loricrin in TNF-α/IFN-γ-induced cells treated with α-boswellic acid was increased, as compared to treatment of TNF-α/IFN-γ cells (Figure 3F–H).

### 2.4. Effects of α-Boswellic Acid on the MAP Kinase Signaling Pathway in TNF-α/IFN-γ-Induced HaCaT Cells

**Figure 4:**
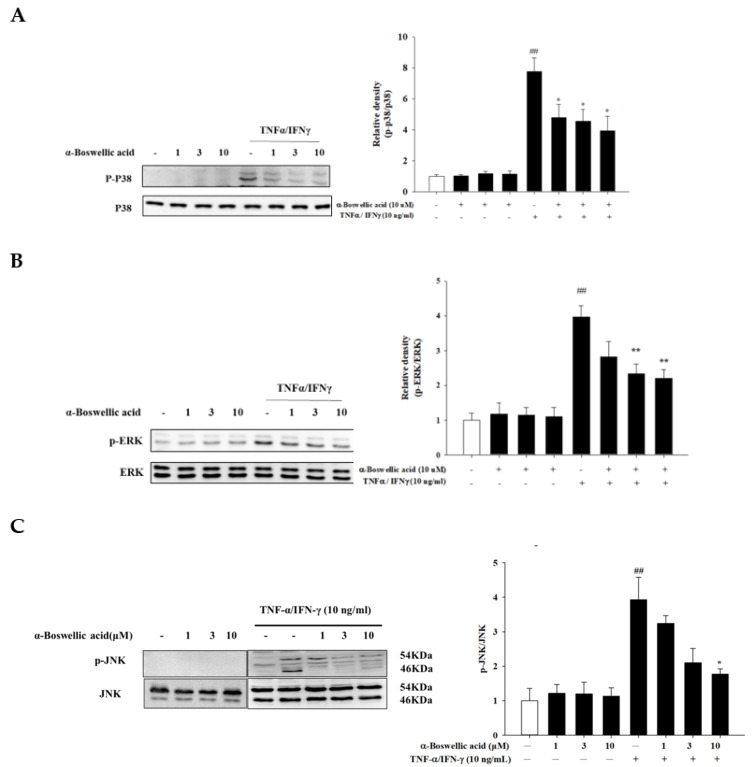
(**A**–**C**) Effects of *α-*boswellic acid on the MAP kinases signaling pathway in TNF-α/IFN-γ-induced HaCaT cells. HaCaT cells were pretreated with different concentrations of α-boswellic acid (1, 3, and 10 μM) for 20 min, and then the cells were treated with TNF-α/IFN-γ for 30 min. Western blots were analyzed quantitatively. Values represent the mean ± SEM from the three independent experiments. ## *p* < 0.01 compared with the no-treatment condition; * *p* < 0.05 and ** *p* < 0.01 compared with the only TNF-α/IFN-γ treatment condition.

The activation of keratinocytes by TNFα/IFNγ leads to the activation of the mitogen-activated protein (MAP) kinases signaling pathway and is closely related to the regulation of various responses throughout the downstream [21]. To investigate the anti-inflammatory mechanism of α-boswellic acid, we determined the expression of the MAP kinases pathways, which are major inflammatory mediators in atopic dermatitis. Western blot was used to analyze the protein phosphorylation of p38, JNK, and ERK. We found that α-boswellic acid potently inhibited ERK, p38, and JNK protein phosphorylation (Figure 4, left panel). Compared to the ratio of total ERK, p38, and JNK levels, p-ERK, p-p38, and p-JNK were significantly reduced (Figure 4, right panel).

### 2.5. Effects of α-Boswellic Acid on the Degradation of IκB and Phosphorylation of NF-κB/p65 Protein in TNF-α-/IFN-γ-Induced HaCaT Cells

**Figure 5:**
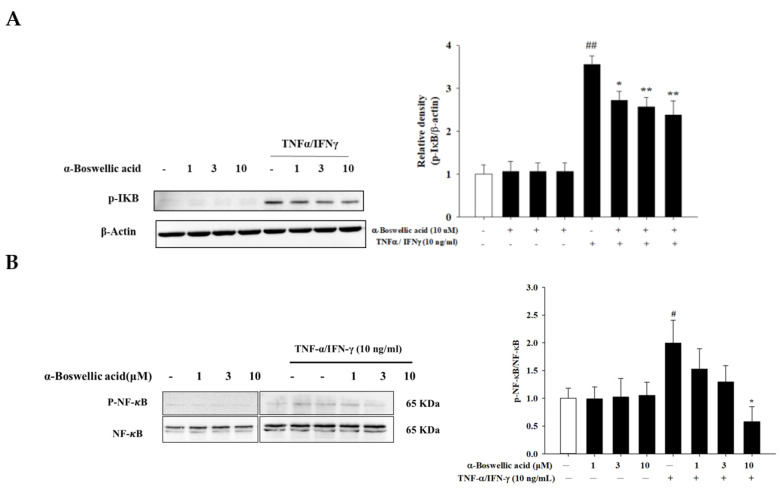
(**A**,**B**) Effects of α-boswellic acid on the degradation of IκB-α and phosphorylation of NF-κB/p65 protein in TNF-α/IFN-γ-induced HaCaT cells. HaCaT cells were pretreated with different concentrations of α-boswellic acid (1, 3, and 10 μM) for 20 min, and then the cells were treated with TNF-α/IFN-γ for 30 min. Western blots were analyzed quantitatively. Values represent the mean ± SEM from three and four independent experiments. # *p* < 0.05 and ## *p* < 0.01 compared with the no-treatment condition; * *p* < 0.05 and ** *p* < 0.01 compared with the only TNF-α/IFN-γ treatment condition.

When HaCaT cells are stimulated by TNF-α/IFN-γ, NF-κB dissociates from IκB and enters the nucleus, thereby promoting the transcription of genes encoding pro-inflammatory factors such as various cytokines. In addition, IκB is phosphorylated and cleaved by the proteasome in the cytosol. In the whole-cell fraction, α-boswellic acid had an inhibitory effect on IκB degradation (Figure 5A, left panel). Relative levels of proteins were calculated as the IκB/β-actin ratio (Figure 5A, right panel). Furthermore, α-boswellic acid effectively inhibited NF-κB phosphorylation in TNF-α/IFN-γ-treated Ha-CaT cells (Figure 5B, left panel). Phosphorylated NF-κB protein intensity was normalized to the total NF-κB protein. As shown in Figure 5B, the right panel shows that α-boswellic acid significantly reduced the phosphorylated NF-κB protein levels. This suggests that α-boswellic acid reduces the expression of pro-inflammatory cytokines by attenuating the effects of TNF-α/IFN-γ-activated MAP kinase and NF-κB signaling pathways in HaCaT cells.

## 3. Discussion

Atopic dermatitis is also the first step in the so-called “atopic march”, with nearly 80 percent of patients later developing asthma or allergic rhinitis [22]. Their common feature is pro-inflammatory mediators from activated immune cells [23]. Treatment with immunosuppressive corticosteroids is currently the most convenient, effective, and cheapest mode of treatment [24]. However, long-term use of corticosteroids can lead to serious side effects [25]. Therefore, finding better alternative treatments is indispensable. With ongoing research, it is a promising and feasible method to find new natural chemicals with fewer side effects from medicinal plants to treat AD [26,27,28]. In the past, we have researched and analyzed plant components with anti-inflammatory and antioxidant properties. Indeed, we found that they have very good anti-atopic dermatitis effects, such as neferine and cycloheterophyllin [29,30,31]. These research results can become the chemical structure basis for the development of effective drugs for the treatment of atopic dermatitis in the future. As a historically used natural product, we found α-bolswellic acid to be a compound with very good anti-atopic dermatitis effects. In skin keratinocytes, it can produce anti-inflammatory effects by inhibiting the production of related inflammatory cytokines.

There are two explanations for the cause of AD: the inside-out hypothesis and the outside-in hypothesis [32]. The inside-out hypothesis is that allergy triggers lead to a weakened skin barrier, which facilitates the introduction and presentation of allergens. This suggests that inflammation is the culprit behind a compromised skin barrier, leading to increased penetration of allergens and microorganisms that elicit responses. The outside-in hypothesis is that a compromised skin barrier precedes AD and is required for immune dysregulation to occur. For example, downregulation of filaggrin (FLG), which is required for skin barrier function, may make the skin more susceptible to immune dysregulation and lead to AD [33]. From our initial findings above, it was discovered that α-boswellic acid alleviates skin scaling, redness, and hyperplasia effects on atopic dermatitis-like skin appearance. It is clear from the appearance that α-boswellic acid is effective. Physiological tests also found that atopic dermatitis-like skin had significantly reduced transepidermal water-loss capacity. It shows that whatever the skin barrier deficiency that causes atopic dermatitis, it can be corrected by α-boswellic acid. Whether these effects are due to the expression or production of proteins constituting the skin barrier, such as filaggrin, is a very important subject for future research [30]. In our preliminary experiments, we did find that cytokines affect the gene expression of structural proteins. It can indeed be restored to normal by the addition of boswellic acid (Figure 3F–H). In our in vitro results, we did see the activation of MAP kinase signaling by cytokines (TNF-α and IFN-γ). It was indeed affected by α-boswellic acid. This signaling pathway is also the most important pathway for atopic dermatitis to produce inflammatory responses. These studies fully understand the anti-inflammatory effect of α-boswellic acid in the skin. In fact, they can be regarded as the main cause of the anti-atopic dermatitis effect of α-boswellic acid. In our main research results, were found the cytokine RNA expression induced by cytokine stimulation was indeed reduced by α-boswellic acid. The effects of these signaling may explain various anti-atopic dermatitis effects in the future. On the other hand, we seem to see splenomegaly and pruritus in atopic dermatitis-like mice that appear to be less affected by α-boswellic acid (results not shown). This result implies that α-boswellic acid has a relatively limited role in some allergic response pathways (such as IgE, etc.), which needs to be proved in the future.

As mentioned earlier, newer treatment strategies for atopic dermatitis focus on improving skin barrier function and targeting polarized immune pathways [34]. Keratinocytes are the main cells constituting the skin-epidermal barrier and play a key role in the pathogenesis of AD. When keratinocytes are damaged by repeated stimuli, such as scratching behaviors, they secrete various keratinocyte-derived cytokines and chemokines that promote the progression of inflammatory skin diseases [32,33]. These secreted cytokines also generate additional responses to AD inflammatory skin lesions by recruiting immune cells such as neutrophils, monocytes, and T cells. T cells recruited to AD inflammatory skin lesions secrete various cytokines, such as IL-4, IL-5, and IL-6, and chemokines, such as IL-8, CCL17, and CXCL10. This secretion of pro-inflammatory cytokines and chemokines is known to result from the activation of various signaling pathways [35]. Based on these mechanisms, we investigated the anti-inflammatory effects of α-boswellic acid on TNF-α/IFN-γ-stimulated keratinocytes and DNCB-induced AD-like skin lesions in mice. HaCaT cells are used in many dermatological studies because they can mimic AD symptoms in response to inflammatory stimuli such as TNF-α/IFN-γ [36]. In AD pathogenesis, keratinocytes have similar roles in the immune response to AD by stimulating TNF-α and IFN-γ [37,38]. In addition, HaCaT cells co-stimulated with TNF-α and IFN-γ have been widely used in AD-related research [39,40]. In this study, we observed that α-boswellic acid did not induce toxicity in HaCaT cells and significantly inhibited the pro-inflammatory cytokines (IL-1β and IL-6) and chemokines (IL-8) in TNF-α/IFN-γ-stimulated HaCaT cells, as well as increased the mRNA levels. In addition, the activation of keratinocytes by TNFα/IFNγ leads to the activation of the MAP kinases signaling pathway and is closely related to the regulation of various responses throughout the downstream [21]. These results suggest that α-boswellic acid has anti-inflammatory and protective effects on cells by inhibiting the production of pro-inflammatory cytokines and chemokines. The intermediate may inhibit the production of inflammatory substances by inhibiting the MAP kinases. On the other hand, transcription factors in the nucleus are important molecules responsible for the transcription of many peptides. NF-κB and STATs are important transcription factors regulating the expression of these inflammatory mediators [41,42]. Among the cytokines and chemokines, the transcription of IL-8 and IL-6 is modulated through an NF-κB element and IL-6 activates the tyrosine phosphorylation of STATs [43]. Therefore, it can be inferred from our present experimental results that α-boswellic acid may reduce the production of IL-6 and IL-8 by inhibiting the phosphorylation of NF-κB. Whether or not it can reduce the formation of cytokine 8 through the inhibition of STATs will be explored and researched in the future.

## 4. Materials and Methods

### 4.1. Reagents

All reagents were purchased from Sigma-Aldrich (St. Louis, MO, USA) unless otherwise noted. The primary anti-bodies anti-p38, anti-ERK1/2, anti-JNK, anti-phospho-ERK1/2, anti-phospho-p38, and anti-phospho-JNK were purchased from Cell Signaling Technology (Beverly, MA, USA). The secondary antibodies were also purchased from Cell Signaling Technology. A Total RNA Isolation Kit (GeneDireX^®^, Vegas, NV, USA), an iScript™ cDNA Synthesis Kit (Bio-Rad, Hercules, CA, USA), and PowerUp™ SYBR™ Green Master Mix (Applied Biosystems™, Waltham, MA, USA) were used for Quantitative Polymer Chain Reaction (PCR) testing. A Pierce Protein Assay Kit (Pierce, Rockford, IL, USA) was used for the Western blot assay.

### 4.2. Cell Culture

The HaCaT cells (Cat. No. CSC-C8977H) were purchased from Creative Bioarray (Shirley, NY, USA), with an STR profiling test. Cells were cultured in DMEM supplemented with 10% fetal bovine serum, 100 units/mL penicillin, and 0.1 mg/mL streptomycin at 37 °C in a humidified environment. All reagents used in the media were purchased from Gibco-BRL (Grand Island, NY, USA). After reaching 80~90% confluence, cells (1 × 10^4^ or 1 × 10^5^ cells/mL) were seeded in 96-well or 24-well culture plates, respectively, and the following experiments were performed.

### 4.3. MTT Assay

The cell suspension (1 × 10^5^ cells/mL) was cultured in 24-well plates for 24 h. Cells were pretreated with or without α-boswellic acid (1–50 μM) for 1 h, then MTT solution was added and incubated for 4 h. Finally, 200 μL of DMSO was added to each well, and 180 μL was pipetted into 96 wells for absorbance measurement. Absorbance was measured at 550 nm using Tecan’s Sunrise absorbance microplate reader (Tecan Trading AG, Männedorf, Switzerland).

### 4.4. mRNA Expression Assay

Total RNA was extracted using a total RNA isolation kit (GeneDireX^®^, Vegas, NV, USA) according to the manufacturer’s instructions. cDNA was synthesized from 1 μg of RNA using the iScript™ cDNA Synthesis Kit (Bio-Rad, Hercules, CA, USA). Real-time PCR was performed using PowerUp™SYBR™Green Master Mix (Applied Biosystems™, Waltham, MA, USA). PCR was performed using the following parameters: 95 °C for 10 min, followed by 40 cycles of 95 °C for 30 s, 55 °C for 30 s, and 72 °C for 45 s. Gene expression was normalized to GAPDH mRNA. The primers used in this study were as follows: for GAPDH, 5′-CTG CTC CTC CTG TTC GAC AGT-3′ and 5′-CCG TTG ACT CCG ACC TTC AC-3′; for IL-6, 5′-CGA GCC CAC CGG GAA CGA AA-3′ and 5′-GGA CCG AAG GCG CTT GTG GAG’; for IL-1β, 5′-CTC TCA CCT CTC CTA CTC ACT-3′ and 5′-ATC AGA ATG TGG GAG CGA AT-3 ‘; For IL-8, 5′-ACT GAG AGT GAT TGA GAG TGG AC-3′ and 5′-AAC CCT CTG CAC CCA GTT TTC-3′; for involucrin, 5′-CAGCAGTCATGTGCTTTTCCT-3′ and 5′-TCCTCCAGTCAATACCCATCAG-3′; for loricrin, 5′-GAGTTGGAGGTGTTTTCCAGGG-3′ and 5′-GCAGAACTAGATGCAGCCGGA-3′; for filaggrin, 5′-TCTGAAGAACCCAGATGATCCA-3′ and 5′-CATCAAAAGAAACTCAGTAAAGTCCAA-3′; for MDC, 5′-GTTGTCCTCGTCCTC CTT GC-3′ and 5′-GGAGTCTGAGGTCCAGTAGAAGTG-3′; for TARC, 5′-GTCTTGAAGCCT CCTCACCC-3′ and 5′-GGATCTCCCTCACTGTGGCT-3′.

### 4.5. Western Blotting Analyses

After scraping, cells were crushed by sonication and centrifuged (13,200 rpm, 10 min, 4 °C). Protein concentrations were determined using Pierce Protein Assay Kit (Pierce, Rockford, IL, USA). Equal amounts of protein were run on 10% SDS-polyacrylamide gels, transferred to nitrocellulose membranes (Millipore, MA, USA) and treated with the anti-bodies anti-p38, anti-ERK1/2, anti-JNK, anti-phospho-ERK1/2, anti-phospho-p38, and anti-phospho-JNK at a 1:1000 ratio. Finally, after adding the secondary antibody for 1 h (diluted to 1:1000), the PVDF membrane was washed 3 times with TBS-T (Tris-buffered saline/0.05% tween 20), then the developing solution was added, and the membrane was put into the chemiluminescence extraction system (BIOSTEP Celvin^®^ S420, Burkhardtsdorf, Germany) for photography.

### 4.6. Animal

**Figure 6:**
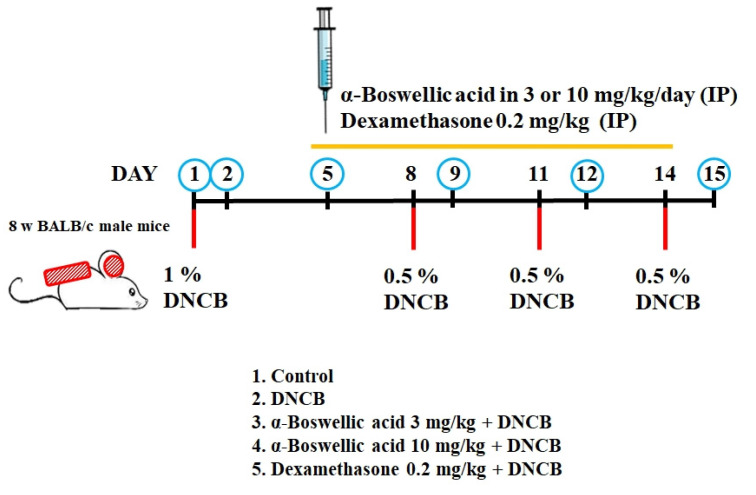
Schematic diagram of the animal experiment. Animal grouping experiments included 1. the normal control group, 2. AD model group (DNCB solution sensitization challenge), 3. and 4. α-boswellic acid group (3 and 10 mg/kg), and 5. positive control group dexamethasone (0.5 mg/kg).

The mice used in the experiment were purchased from the National Laboratory Animal Center in Taiwan. First, the effect of α-boswellic acid on AD was assessed on DNBC-induced mice. DNBC-induced AD was developed according to a previously reported method [29]. The dorsal hairs of the mice were briefly shaved and then 100 μL and 20 μL of 1% DNCB in ethanol were applied to sensitive dorsal and ear skin for 7 days. Then, from Day 8 to Day 15, 0.5% DNCB solution was re-challenged every three days. The schematic diagram of the animal experiment is shown in Figure 6. Mice were randomly divided into 5 groups, including the normal control group, AD model group (DNCB solution sensitization challenge), α-boswellic acid group (3 and 10 mg/kg), and positive control group dexamethasone (0.5 mg/kg). α-Boswellic acid or dexamethasone was injected intraperitoneally every 3 days from Day 5 to Day 15. On Day 15 before sacrifice, the lesions on the back skin of the mice were photographed, including the erythema, edema, crusting, and exfoliation. The dorsal skin was isolated, the skin tissue port was fixed in 4% paraformaldehyde for histological analysis, and the remaining skin tissue was immediately frozen in liquid nitrogen and then stored at −80 °C for subsequent studies.

### 4.7. Statistical Analyses

Data are presented as the mean ± SEM, with (*) and (#) as notes. Student’s *t*-test for two-group comparisons and one-way ANOVA were used, followed by post-hoc tests for multiple comparisons among more than two groups. Differences were considered statistically significant at *p* < 0.05. Sigma-Plot software (Version 14.0) was used for the statistical analysis.

## 5. Conclusions

This study demonstrates the therapeutic potential of α-boswellic acid for AD and the underlying mechanisms in vivo and in vitro. Our current research results show that α-boswellic acid can effectively improve the erythema, abrasion, and desquamation, as well as repair the dysfunctional skin barrier, reduce inflammation, reduce mast cell infiltration, reduce MAP kinase expression, and inhibit the NF-κB pathway in AD model mouse skin and TNF-α/IFN-γ-stimulated HaCaT cells. In conclusion, our study provides a basis for the future application of α-boswellic acid in the treatment of AD by inhibiting the production of inflammatory mediators and restoring the skin barrier.

## Data Availability

Not applicable.
